# High-affinity omalizumab variants with optimized disruptive potency prevent anaphylaxis *in vivo*

**DOI:** 10.1016/j.jaci.2025.05.028

**Published:** 2025-10-13

**Authors:** Daniel Brigger, Pascal Guntern, Luke F. Pennington, Robin van Brummelen, Theodore S. Jardetzky, Alexander Eggel

**Affiliations:** a Department of BioMedical Research, University of Bern, Bern;; b Department of Rheumatology and Immunology, University Hospital Bern, Bern;; c Excellergy Inc, Palo Alto;; d Department of Structural Biology, Stanford University School of Medicine, Stanford.

**Keywords:** IgE, anti-IgE antibody, mast cells, basophils, receptor-ligand disruption, passive systemic anaphylaxis

## Abstract

**Background:**

Omalizumab, a therapeutic mAb targeting IgE, is approved for the treatment of multiple allergic indications. However, its moderate affinity for IgE necessitates frequent high-dose administrations, limiting its therapeutic use and efficacy. Attempts to develop next-generation anti-IgE antibodies with improved affinity, such as ligelizumab or HAE1, have yielded alternatives that are either less safe or not demonstrably superior.

**Objective:**

We sought to generate optimized omalizumab variants featuring 2 specific molecular enhancements: increased IgE binding affinity while preserving epitope specificity to enhance target neutralization and improved potency to actively dissociate prebound IgE from its high-affinity receptor FcεRI. Methods: Using a targeted yeast display selection strategy applied to mutated omalizumab libraries, we identified the anti-IgE clone C03 and engineered 2 flexible variants, C03-H1L2 and C03-H2L2.

**Results:**

The C03 antibodies demonstrated approximately 10-fold higher IgE binding affinity compared with omalizumab, resulting in superior inhibition of IgE binding to FcεRI. Furthermore, C03-H1L2 and C03-H2L2 exhibited enhanced potency in displacing FcεRI-bound IgE from humanized mouse mast cells and human basophils without triggering spontaneous cell activation. In a systemic anaphylaxis mouse model, single-dose administration of the flexible C03 variants, in contrast to omalizumab, desensitized allergic effector cells within 36 hours, fully preventing antigen-induced anaphylaxis.

**Conclusions:**

These findings underscore the importance of engineering next-generation anti-IgE therapies with higher affinity and disruptive potency to optimize current treatment approaches.

## Capsule summary

Next-generation anti-IgE antibodies with high affinity and improved potency to remove IgE from its high-affinity receptor FcεRIα rapidly desensitize allergic effector cells and prevent antigen-mediated anaphylaxis *in vivo*.

## GRAPHICAL ABSTRACT

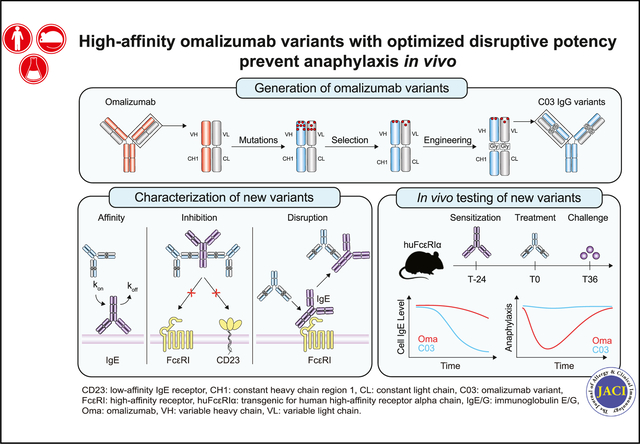

IgE-driven type I hypersensitivity reactions are characterized by an exaggerated immune response to normally harmless environmental substances, such as pollen, dust mites, or food proteins.^1^ The severity of such allergic reactions can manifest with symptoms ranging from moderate local irritations (ie, rhinoconjunctivitis) to fatal multiorgan failure (ie, anaphylaxis). Binding of allergen-specific IgE to its high-affinity IgE receptor, FcεRI, on mast cells (MCs) and basophils is at the core of the allergic pathophysiology, as allergen-mediated cross-linking of FCεRI-bound IgE triggers an intracellular signaling cascade in these cells resulting in the release of prestored and *de novo* synthesized inflammatory mediators (ie, histamine, leukotrienes, prostaglandins, cytokines, chemokines, and proteases).^2,3^ The biological role of the low-affinity IgE receptor FcεRII (also known as CD23) in allergic responses is less clear. However, it has been implicated in the display of allergen peptides to T cells by antigen-presenting cells, the regulation of IgE production in B cells, and the transport of antigens cross epithelial barriers.^4–7^

Omalizumab is a therapeutic anti-IgE mAb that was initially developed as a treatment for allergic asthma,^8,9^ but has since been approved for several other indications, including chronic spontaneous urticaria,^10,11^ nasal polyposis,^12,13^ and food allergy.^14^ Its primary mode of action is the binding and neutralization of free serum IgE. By that means, omalizumab efficiently blocks IgE interactions with both primary receptors, FcεRI and CD23,^15^ and decreases FcεRI surface levels on MCs and basophils owing to receptor destabilization.^16,17^

Recent studies have suggested that omalizumab features additional modes of action beyond binding and neutralization of free serum IgE.^18^ For example, we have demonstrated that it accelerates the removal of FcεRI-bound IgE from the surface of allergic effector cells through a process called facilitated dissociation and thereby actively desensitizes basophils, MCs, and dendritic cells.^19,20^ This mechanism occurs when omalizumab transiently engages the IgE:FcεRI complex, promotes the disengagement of the individual components, and captures IgE in an inhibited state.^21^ Despite the slow kinetics and high antibody concentrations required, omalizumab-induced dissociation of cell surface bound IgE might be of considerable clinical relevance, especially as patients are usually treated over multiple years. Moreover, omalizumab has been shown to effectively inhibit CD23-related biological mechanisms,^20,22,23^ such as the blocking of IgE binding to CD23 on dendritic cells, which reduces their antigen-presenting capabilities.^24^ The totality of these molecular mechanisms paired with its unique epitope specificity has turned omalizumab into an efficient option to treat multiple allergic conditions.^25^

Despite its clinical success, omalizumab has several limitations. The need for regular dosing and long treatment regimen, which are mainly due to its relatively moderate affinity for IgE (fragment antigen-binding [Fab] K_D_ approximately 15 nM), represent 2 major challenges for broader clinical adoption. In addition, the dosing regimen varies among patients depending on their total serum IgE level and body weight. High total serum IgE levels (>700 kU/L in patients >_12 years of age) and body weight (>150 kg) are exclusion criteria for omalizumab therapy in allergic asthma as the required dosage and the associated costs become difficult to justify.^9^ Additionally, the moderate affinity of omalizumab leads to the inability of diagnostic assays to accurately quantify the level of free IgE in the serum of treated patients.^26^ To overcome these challenges, there have been various efforts to develop affinity matured omalizumab variants, including the high-affinity anti-IgE antibody HAE1 (also known as PRO98498 or E27).^27,28^ Early studies have indicated that HAE1, which differs from omalizumab in 9 amino acids at the complementary determining regions, features a 23-fold increased affinity for IgE and is more effective at inhibiting IgE binding to FcεRI on rat basophilic leukemia cells carrying the human receptor *in vitro*.^28^ Preclinical studies with HAE1 in cynomolgus monkeys found that drug exposure for HAE1 and omalizumab were similar, but that HAE1 suppressed free IgE more efficiently at lower doses.^28^ This finding was reproduced in a clinical phase 1 trial in humans, where lower HAE1 doses (90 mg) achieved greater free IgE suppression than regular omalizumab doses (150 mg).^28^ The clinical development program for HAE1 was discontinued after 2 participants experienced adverse events in the phase 2 trial (NCT00406965); however, the exact nature of these events has not been published.

We have previously reported an efficient approach to select potent omalizumab variants using yeast-based, high-throughput screens.^29^ Our structural work has further highlighted that Fab flexibility between the variable heavy chain (VH) and variable light chain (VL) domains of omalizumab-like anti-IgE antibodies can modulate binding affinity and potency of IgE disruption.^29^ Based on these findings and to potentially overcome limitations associated with the current anti-IgE treatment, we sought to generate more potent omalizumab variants that maintain key elements of its pharmacologic profile, including free IgE blockade and lack of effector cell activation, while enhancing affinity to IgE and potency to dissociate IgE:FcεRI complexes.

## METHODS

For detailed information on used recombinant proteins and antibodies, protein interaction measurements with surface plasmon resonance (SPR), ELISAs, cellular assays, passive systemic anaphylaxis model, flow cytometry, and immune complex formation, see this article’s Methods section in the Online Repository available at www.jacionline.org.

### Protein interaction measurements with SPR

All SPR measurements were carried out on a Biacore X100 system (Cytvia, Marlborough, Mass). HBS-EP+ Buffer was used as running buffer at a flow rate of 10 μL/min. The target proteins were immobilized on Fc2 of a Biacore Sensor Chip CM5 by standard amine coupling. The sensorgrams reflect binding responses on Fc2 minus binding responses on the reference Fc1. For some curves, excessive buffer peaks were excluded from the data. To determine binding kinetics, we used the BIAevaluation software. Affinity constants were calculated using a 1:1 langmuir curve fitting model.

### ELISAs

Anti-IgE-mediated blocking of IgE binding to CD23 was assessed by measuring binding of IgE-antigen complexes preincubated with anti-IgE antibodies on immobilized recombinant human CD23. Inhibition of IgE binding to FcεRI was assessed by measuring binding of IgE preincubated with anti-IgE antibodies on immobilized human recombinant FcεRIα. Anti-IgE-mediated disruption of IgE:FcεRI complexes was assessed by measuring remaining receptor-bound IgE after incubation with anti-IgE antibodies. Time-dependent disruption of IgE:FcεRIα complexes was assessed by measuring remaining receptor-bound IgE following incubation with anti-IgE antibodies after different incubation times. Plasma anti-IgE antibody concentrations were determined using the IgG high-sensitivity ELISA.

### Cellular assays

RPMI8866, expressing high levels of human CD23, cells were cultured in RPMI+/+. To assess anti-IgE-mediated blocking of IgE binding to CD23 on RPMI8866 cells, anti-IgE antibodies were mixed with IgE and allowed to complex. The mixture was added to RPMI8866 cells, followed by detection of remaining cell-bound IgE by flow cytometry. Hoxb8 MCs were cultured in RPMI+/+ including IL-3 and 4-hydroxytamoxifen (tamoxifen). A total of 50,000 differentiated Hoxb8 MCs were sensitized with IgE overnight. Cells were washed, and anti-IgE antibodies were added. Remaining cell-bound IgE was quantified by flow cytometry. To measure inhibition of IgE binding to FcεRIα, IgE preincubated with anti-IgE antibodies was added to 50,000 Hoxb8 MCs. Cell-bound IgE was quantified by flow cytometry. Whole blood from voluntary donors was collected at the University Hospital Bern with approval from the local ethics committee of the Canton of Bern. Informed consent was obtained in accordance with the Declaration of Helsinki. Basophil activation test was performed by adding anti-IgE antibodies to the blood. Cell-bound IgE as well as activation of basophils was quantified by flow cytometry. To calculate percentage of IgE removal, background mean fluorescence intensity (autofluorescence of cells) was subtracted, and mean fluorescence intensity was normalized to IgE levels of samples without anti-IgE treatment.

### Passive systemic anaphylaxis mouse model

Mice transgenic for human FcεRIα were kindly provided by Prof. J.-P. Kinet. All animal experimentation was approved by the local ethics committee of the Canton of Bern. Mice were passively sensitized intraperitoneally with antigen-specific IgE. The next day, anti-IgE antibody variants were injected intraperitoneally. Mice were challenged 36 hours after anti-IgE treatment with antigen. Body core temperature was assessed for 2 hours. Blood was drawn at indicated time points to assess IgE surface levels on blood basophils using flow cytometry and to measure anti-IgE antibody concentrations in the plasma fraction. Temperature loss (Δ°C core body temperature) is represented for each time point after subtracting baseline temperature, and area under the curve has been calculated using GraphPad Prism (GraphPad Software, Boston, Mass). Mice were sacrificed, and the peritoneal cavity collected for further analysis.

### Statistical analysis

Nonlinear regression curve analysis was performed for curve fitting and determination of half-maximal inhibitory concentration (IC_50_) or half-maximal inhibitory concentration (DC_50_) in ELISAs and flow cytometry experiments. For the *in vivo* experiment, statistical differences between different treatment groups were calculated using one-way ANOVA with Tukey multiple comparisons against PBS treatment.

## RESULTS

### Omalizumab variants show favorable expression properties and high stability

**FIG 1.**
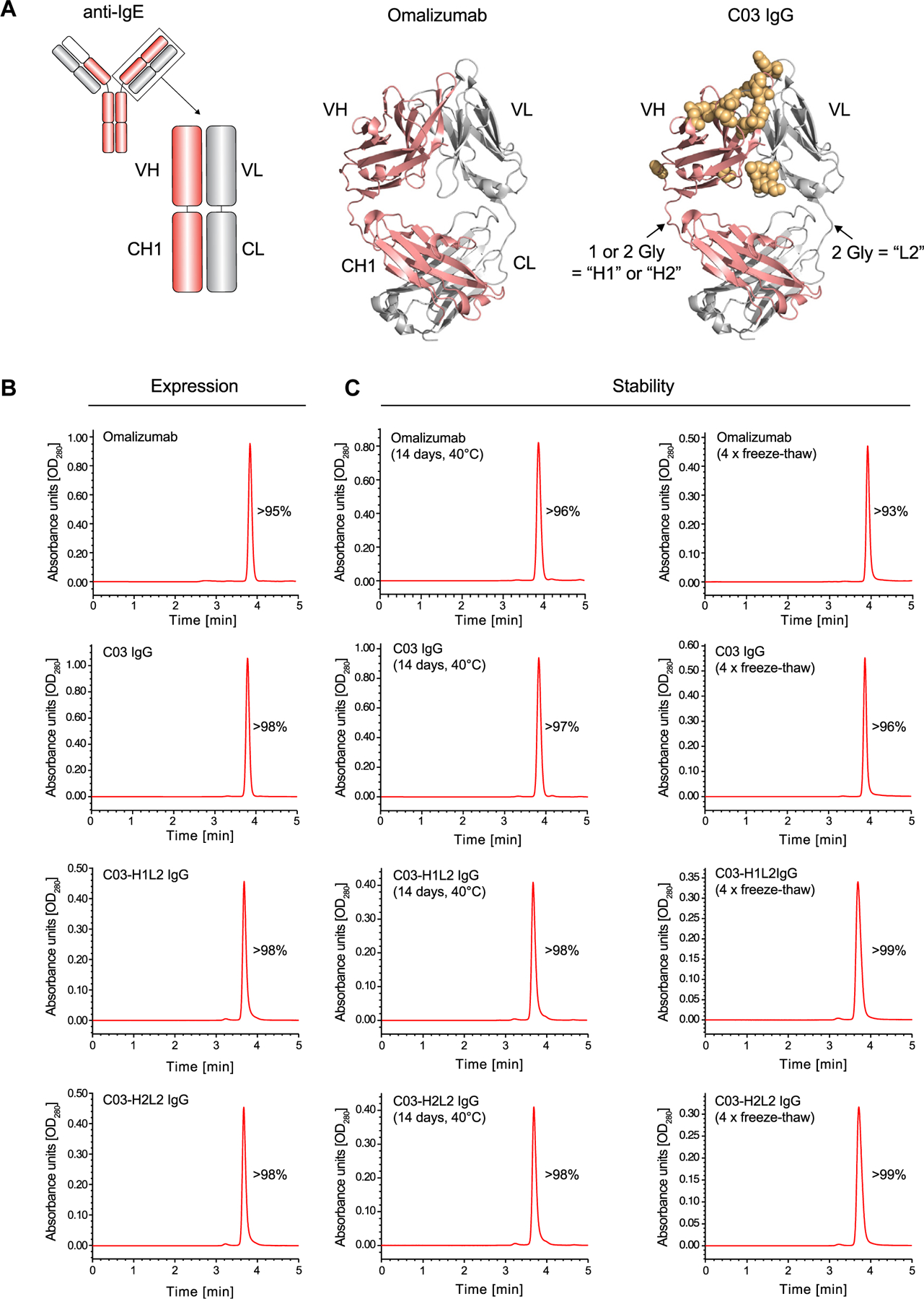
Comparison between omalizumab and the identified C03 variant. **(A)** Drawing of an anti-IgE antibody with a focus on its Fab, consisting of a heavy *(red)* and light *(light gray)* chain with a constant and a variable region *(left panel)*. Crystal structure of omalizumab Fab (Protein Data Bank: 5hys) with the heavy *(red)* and light *(gray)* chain as reference *(middle panel)*. Positions of selected amino acid mutations in the antibody variant C03 *(light brown spheres)* compared with omalizumab *(right panel)*. Gly insertions between the constant and variable domains of the heavy (1 or 2 Gly) and light (2 Gly) chain are indicated. **(B)** SEC histograms of recombinantly produced omalizumab and C03 antibody variants including the percentage of monomeric peak content are shown *(left panel)*. Histograms of the anti-IgE antibodies after stress testing for 14 days at 40°C *(middle panel)* or for 4 sequential freeze-thaw cycles *(right panel)*. Differently scaled y-axes were plotted in the histograms.

Using our previously described yeast display strategy,^29^ we screened omalizumab mutant libraries and identified antibody clone C03. Compared with omalizumab, C03 carries >10 mutations in the light and heavy chain complementary determining regions, the antibody framework, and at the VH/VL interface (Fig 1, A). Additionally, we expressed the parental C03 antibody as flexible variants with either 1 or 2 glycine (Gly) insertions at the interface of the VH/VL domains and the corresponding constant region (CH_1_) in each Fab (Fig 1, A). In recombinant expression as IgG1 format, all C03 antibodies showed high yields with >98% monomer content as measured by size exclusion chromatography (SEC) (Fig 1, B). To assess the impact of Gly insertion on antibody stability, we assessed the melting and aggregation temperatures of each antibody by differential scanning fluorimetry and static light scattering, respectively. Melting temperature indicates the temperature at which 50% of the antibody unfolds, representing its structural or thermal stability, whereas aggregation temperature marks the onset of aggregation, reflecting its stability in solution. Whereas the Gly insertions in C03-H1L2 and C03-H2L2 led to a small decrease in thermal stability compared with the parental C03 antibody, all antibodies retained a melting temperature >60°C (Table E1 in the Online Repository at www.jacionline.org). To assess the impact of these minor changes in thermal stability on long-term storage and freeze-thaw behavior, we additionally incubated the antibodies for 14 days at 40°C or performed 4 consecutive freeze-thaw cycles. All C03 variants showed favorable stability profiles and retained >96% monomer content (Fig 1, C).

### Omalizumab variants recognize human and macaque IgE with high affinity and form stable IgE:anti-IgE complexes

**FIG 2.**
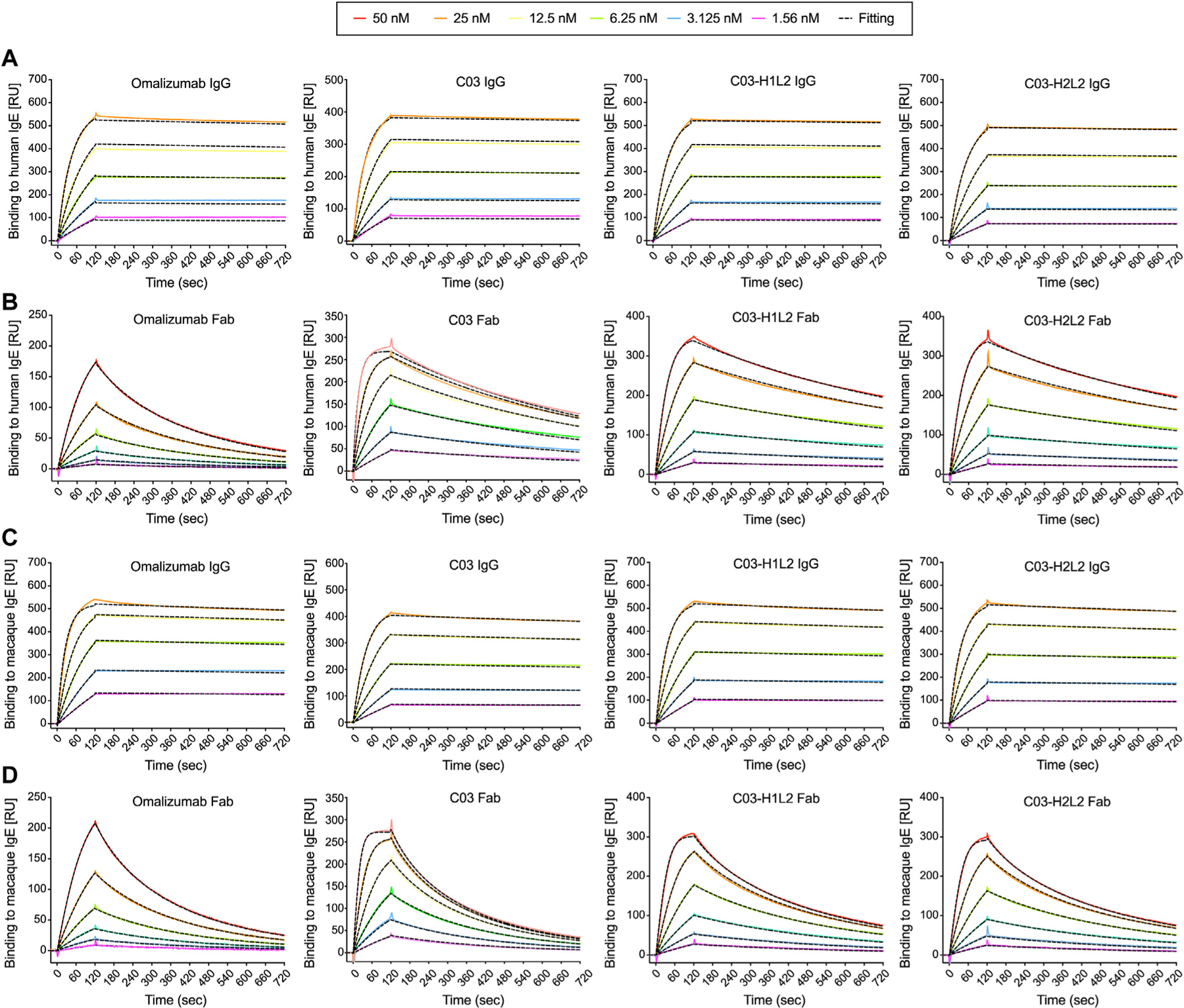
Binding kinetics of anti-IgE antibodies to recombinant human and macaque IgE. Histograms displaying association and dissociation curves of omalizumab, C03, C03-H1L2, and C03-H2L2 full-length IgG **(A** and **C)** and Fab fragments **(B** and **D)** to recombinant human **(A** and **B)** and macaque **(C** and **D)** IgE by SPR are shown. Each color refers to an individual measurement cycle at a given antibody concentration (1.56–25 nM or 1.56–50 nM). Curves were fitted *(black dashes)* using a 1:1 langmuir binding model. Differently scaled y-axes were plotted in the sensorgrams. *RU*, Response units.

We next measured binding kinetics of the C03 variants and omalizumab to human and macaque full-length IgE using SPR. Different concentrations of anti-IgE antibodies in either full-length or Fab fragment format were injected to recombinant IgE (trastuzumab-IgE; anti-Her2-IgE), which was captured via covalently immobilized Her2 on the chip surface. Whereas the association phase was measured for 120 seconds, the dissociation phase of the formed complexes was observed over 300 seconds (Fig 2, A–D). The fitted association constant (k_a_) and dissociation constant (k_d_) resulting from a 1:1 langmuir binding model analysis were used to calculate the equilibrium dissociation constant (K_D_) for each anti-IgE antibody and Fab fragment (Table I). All full-length anti-IgE antibodies showed very similar high-affinity binding in the picomolar range to both human and macaque IgE. This result is likely due to the avidity effect driven by antibody bivalency and the detection limitation of the device. The real difference in affinity became apparent in the monovalent Fab fragment formats of the anti-IgE antibodies (Table I), in which parental C03 Fab (K_D_ 1.1 nM), C03-H1L2 Fab (K_D_ 1.4 nM), and C03-H2L2 Fab (K_D_ 1.5 nM) all showed a >_10-fold improvement over omalizumab Fab (K_D_ 15 nM). The observed affinity gain of the C03 variants was mainly driven by slower off-rates. Interestingly, parental C03 Fab (K_D_ 3.3 nM), C03-H1L2 Fab (K_D_ 3.3 nM), and C03-H2L2 Fab (K_D_ 3.5 nM) showed only an approximately 4.5-fold affinity gain over omalizumab Fab for macaque IgE, whereas the affinity of omalizumab Fab remained unaltered (K_D_ 16 nM) compared with human IgE. The data indicate that the Gly insertions between the VH/VL domains and the constant region of C03 did not affect overall affinity of the antibodies and that the new C03 antibody variants exhibited significantly higher affinity than omalizumab.

As previously described for omalizumab and ligelizumab, the size and stoichiometry of immune complexes formed between free IgE and different anti-IgE antibodies may vary considerably depending on affinity and epitope specificity.^18^ The resulting drug:IgE immune complexes that accumulate in circulation lead to an increase in total serum IgE and prolong the serum half-life of IgE during treatment. This effect is driven by the relatively slow clearance of drug:IgE complexes compared with free IgE, which has a short serum half-life of 2 to 3 days.^9,30^ Besides the neutralization of IgE and the inhibition of its binding to the surface of allergic effector cells, omalizumab:IgE complexes can act as allergen sweepers that prevent allergens from reaching IgE on allergic effector cells.^31^ Here, we compared complex formation between recombinant human trastuzumab-IgE (molecular weight: approximately 190 kDa) and omalizumab or C03 variants (molecular weight: approximately 150 kDa) at different molar ratios by SEC with multiangle light scattering. Although the SEC histograms looked slightly different for each anti-IgE, the resulting immune complex distribution was remarkably similar (Fig E1 in the Online Repository at www.jacionline.org). All tested anti-IgE antibodies showed a binding stoichiometry ranging from 1:1 up to maximally 4:4. At an excess of anti-IgE or at equimolar concentrations, complexes preferentially occurred at 3:3 binding stoichiometry for omalizumab, with increasing abundance of 2:2 complexes seen in the higher-affinity C03 variants, respectively. Neither omalizumab nor the 2 C03 variants showed an abundance of higher-molecular-weight aggregates. Based on these data, similar clearance rates as previously reported for omalizumab^32^ would be expected for C03-H1L2 and C03-H2L2.

### Omalizumab variants are superior at inhibiting IgE:FcεRIα, but not IgE:CD23, interactions

**FIG 3.**
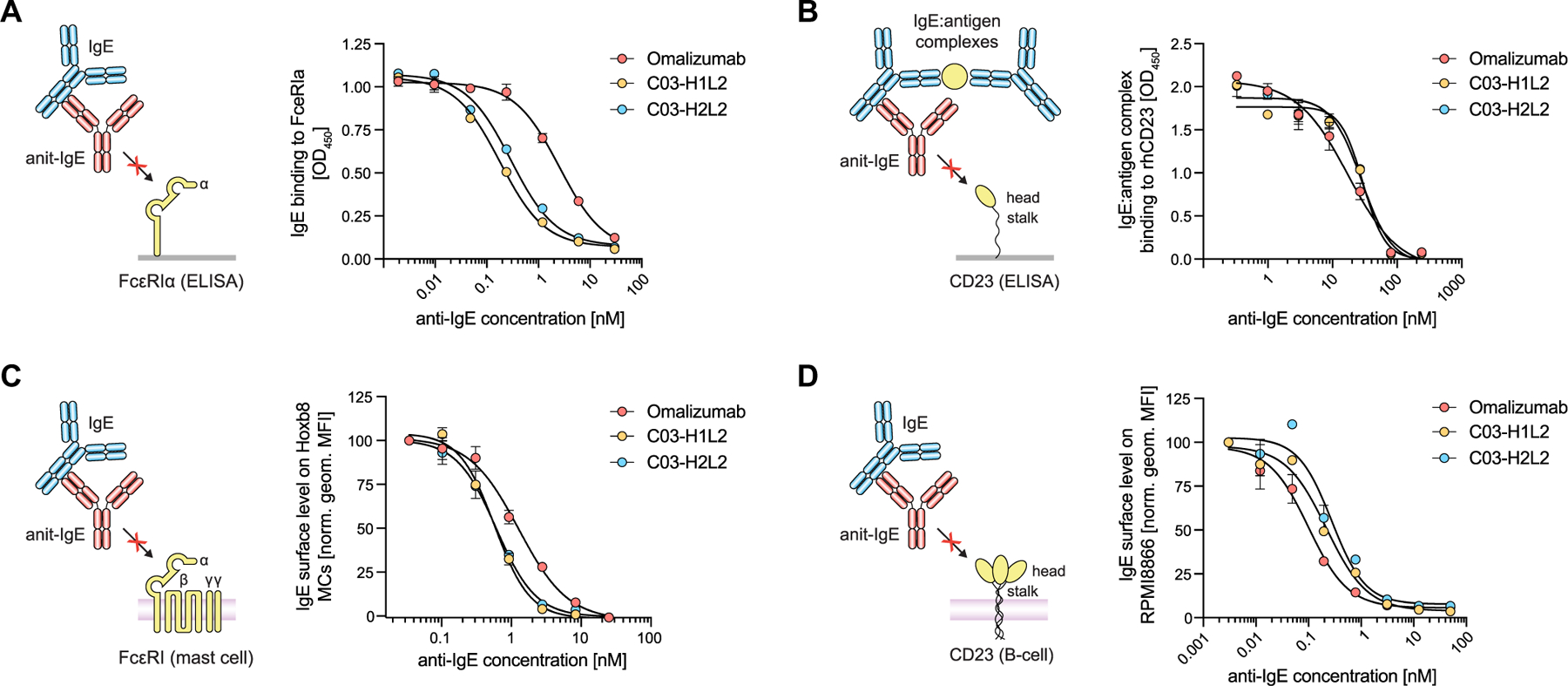
Inhibition of IgE binding to human FcεRIα and CD23. **(A)** Inhibition of IgE binding to FcεRIα by different concentrations (0.002–30 nM) of omalizumab *(red)*, C03-H1L2 *(orange)*, or C03-H2L2 *(blue)* full-length IgGs as assessed by ELISA. **(B)** Inhibition of IgE:antigen complex binding to CD23 by different concentrations (0.3–240 nM) of omalizumab, C03-H1L2, or C03-H2L2 full-length IgGs as measured by ELISA. **(C)** Normalized inhibition of 1 nM IgE binding to FcεRIα on Hoxb8 MCs by different concentrations (0.3–25 nM) of omalizumab, C03-H1L2, and C03-H2L2 full-length IgGs as measured by flow cytometry. **(D)** Normalized inhibition of IgE binding to CD23 on RPMI8866 cells by different concentrations (0.003–50 nM) of omalizumab, C03-H1L2, and C03-H2L2 full-length IgGs as measured by flow cytometry. All inhibition curves were fitted using a nonlinear regression model *(black lines)*.

Given that C03-H1L2 and C03-H2L2 showed higher monomeric affinity for IgE compared with omalizumab, but similar picomolar binding as intact IgG format, we assessed their potency to inhibit IgE binding to FcεRIα by ELISA (Fig 3, A). Recombinant biotinylated humanized IgE (0.04 nM 3-nitro-4-hydroxy-5-iodophenylacetyl [NIP]–specific JW8-IgE) was preincubated with different concentrations of anti-IgE (0.002–30 nM) for 30 minutes and were subsequently added to immobilized recombinant FcεRIα for 1 hour. The amount of receptor-bound IgE was detected with streptavidin-conjugated horseradish peroxidase. Compared with omalizumab (IC_50_: 2.529 nM), both C03 variants showed approximately 10-fold superior inhibition (IC_50_: C03-H1L2 0.1810 nM; C03-H2L2 0.2927 nM). A similar blocking assay was performed with recombinant CD23, in which NIP-BSA–complexed biotinylated JW8-IgE (240 nM; 1:1 ratio) was preincubated with different concentrations of anti-IgE (0.3–240 nM) for 30 minutes and subsequently added to CD23 for 1 hour (Fig 3, B). CD23-bound IgE was detected with streptavidin-conjugated horseradish peroxidase. All 3 antibodies showed a similar dose-dependent inhibition curve with minor (<2-fold) changes in efficacy (IC_50_ omalizumab 17.48 nM; C03-H1L2 29.65 nM; C03-H2L2 28.24 nM).

We further compared the IgE neutralizing capacity of all 3 anti-IgEs in a cell-based assay. For this purpose, we used the Hoxb8 MC line expressing high levels of human FcεRIα.^33,34^ A fixed concentration (1 nM) of biotinylated recombinant human IgE (Sus11-IgE^35^) was preincubated for 30 minutes with varying concentrations of the anti-IgE antibodies (0.3–25 nM). These antibody mixtures were added to the cells for 1 hour and the amount of cell surface bound IgE was quantified by flow cytometry (Fig 3, C; Fig E2, A, in the Online Repository at www.jacionline.org). Although both omalizumab variants showed lower IC_50_ concentrations for the inhibition of IgE binding to FcεRIα, the observed differences were less pronounced than in the ELISA (IC_50_ C03-H1L2: 0.5764 nM < C03-H2L2: 0.6070 nM < omalizumab: 1.271 nM). Interestingly, in this assay the inhibitory differentiation between the three antibodies decreased with increasing IgE concentrations (Fig E2, B). This indicates that the IC_50_ of neutralization more closely relates to the stoichiometric relationship of anti-IgE to IgE, rather than the monomeric affinity of a given anti-IgE. We additionally assessed the inhibitory efficacy of all three antibodies on the CD23 expressing B-cell line RPMI8866 (Fig 3, D). Monomeric IgE (12.5 nM JW8-IgE) was preincubated with different concentrations of anti-IgE (0.003–50 nM) for 30 minutes and subsequently added to the cells for 1 hour. CD23-bound IgE was detected using an APC-conjugated anti-mouse lambda light chain antibody by flow cytometry (Fig E2, C). As previously published, our results confirmed that omalizumab features a remarkably high potency to inhibit IgE-binding to CD23.^15^ Both high-affinity omalizumab variants showed only a minor loss of inhibitory potency (IC_50_ omalizumab: 0.0995 nM < IC_50_ C03-H1L2: 0.2197 nM < IC_50_ C03-H2L2: 0.2763 nM). Overall, these results were largely in line with the ELISA data, showing superior inhibition of IgE:FcεRIα and slightly less potent blocking of IgE:CD23 interactions by C03 antibody variants.

### Flexible omalizumab variants feature improved disruptive efficacy

**FIG 4.**
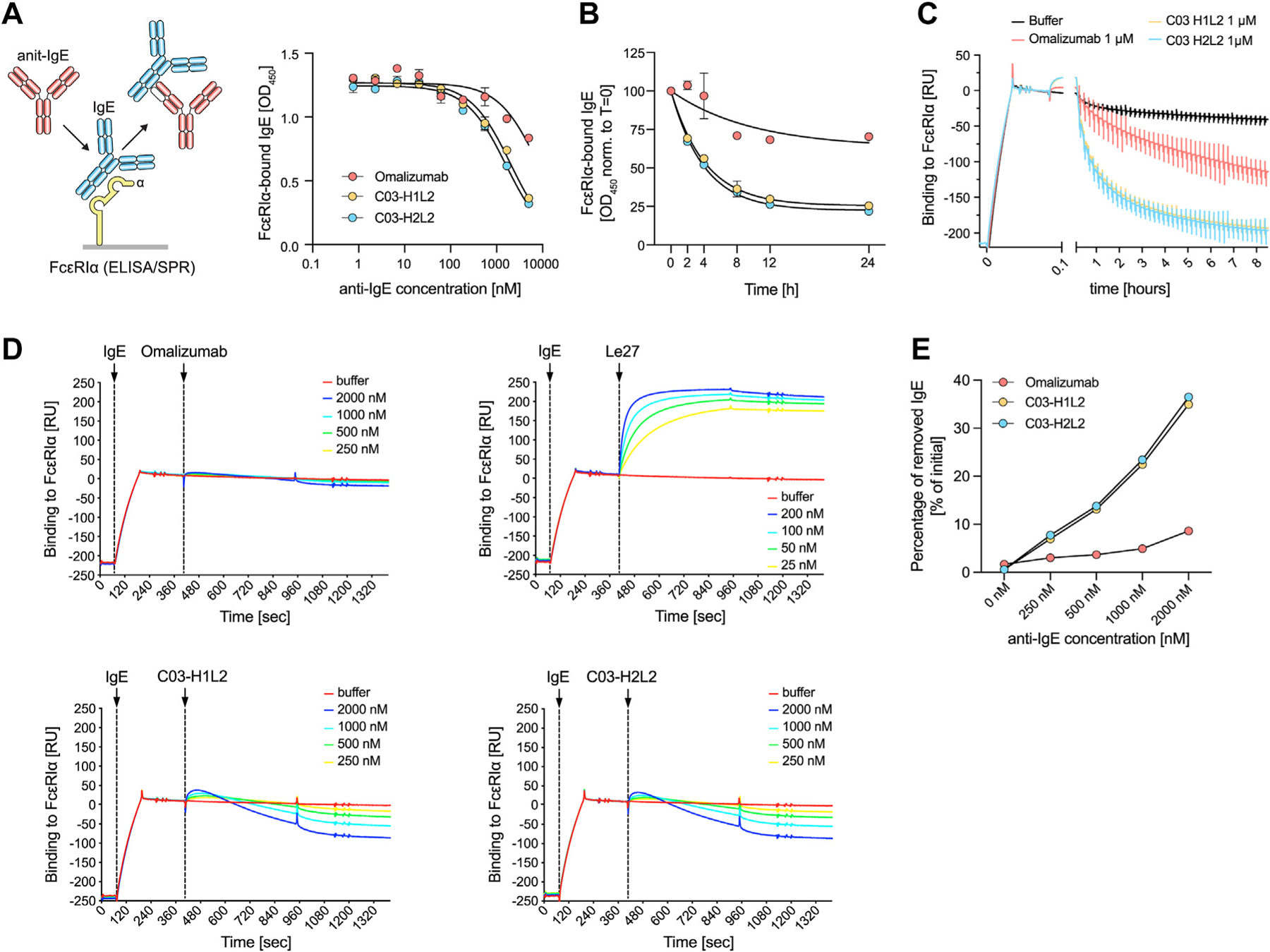
Molecular disruption kinetics assessment of anti-IgE antibodies assessed by ELISA and SPR. Dose-dependent (0.0008–5 μM) **(A)** or time-dependent (0, 2, 4, 8, 12, and 24 hours at 1 μM) **(B)** disruption of preformed IgE:FcεRIα complexes on omalizumab *(red)*, C03-H1L2 *(orange)*, and C03-H2L2 *(blue)* full-length IgG incubation by ELISA. **(C)** Real-time assessment of IgE removal from surface immobilized FcεRIα using 1 μM of omalizumab, C03-H1L2, or C03-H2L2 over 8 hours compared with buffer flow *(black)* by SPR. **(D)** Short-term real-time assessment of IgE removal from surface immobilized FcεRIα using different concentrations (0.25–2 μM) of omalizumab, C03-H1L2, or C03-H2L2 over 1400 seconds compared with buffer flow *(red)* by SPR. Each color refers to an individual measurement cycle. *Black arrows* indicate time of injection. **(E)** Quantification of percentage IgE removed from surface immobilized FcεRIα during short-term incubation across different concentrations of omalizumab *(red)*, C03-H1L2 *(orange)*, and C03-H2L2 *(blue)* full-length IgG. Data of technical duplicates are shown as mean ± SEM **(A** and **B)**. *RU*, Response units.

Previously, we demonstrated that omalizumab, in addition to its primary mode of action of neutralizing free serum IgE, has the ability to accelerate the dissociation of IgE from FcεRIα at high concentrations.^19^ To test whether the C03-H1L2 and C03-H2L2 feature improved disruptive efficacy, we first performed an ELISA in which we preformed FcεRIα:IgE complexes on solid phase. Increasing concentrations of the 3 anti-IgE antibodies (0.0008–5 μM) were subsequently added for 12 hours (Fig 4, A). After washing of the plate, the amount of remaining FcεRIα-bound IgE was measured. Both C03-H1L2 and C03-H2L2 showed higher disruptive potency (DC_50_) than omalizumab (DC_50_ omalizumab: 8027 nM; DC_50_ C03-H1L2: 2007 nM; DC_50_ C03-H2L2: 1609 nM). Importantly, the observed disruption was not only dose dependent, but also time dependent, as demonstrated in an additional ELISA, in which preformed FcεRIα:IgE complexes were incubated with a fixed concentration of anti-IgE (5 μM) over 2, 4, 8, 12, and 24 hours (Fig 4, B). To further corroborate these findings and get real-time evidence for the removal of IgE from FcεRIα, we performed SPR experiments (Fig 4, C and D). Solid-phase immobilized FcεRIα was exposed to recombinant human IgE (Sus11-IgE) to reach a final capture response of >200 response units. Subsequently, repeated injections of anti-IgE antibodies at a single concentration (1 μM) were performed for approximately 8 hours (Fig 4, C), or a single injection at different concentrations (250–2000 nM) was performed for 9 minutes (Fig 4, D). Only minor intrinsic FcεRIα:IgE dissociations were observed in buffer control runs owing to high-affinity receptor-ligand interaction. The drop in response curves on anti-IgE injection again confirmed time- and dose-dependent removal of IgE. Long-term anti-IgE exposure led to almost complete removal of IgE from the receptor by C03-H1L2 and C03-H2L2, whereas omalizumab dissociated only approximately 50% of the initially bound IgE during that time. In the short-term experiment, the distinct binding profiles of different anti-IgE antibodies became even more evident. As expected, nondisruptive anti-IgE antibodies such as Le27 readily bound to FcεRIα-bound IgE and formed tertiary complexes as seen in the immediate increase of the response curve. Omalizumab showed only minor dissociation with <10% removal of the initial amount of receptor-bound IgE at the highest concentration in this short time frame. The flexible C03 antibodies, however, removed >30% under these conditions (Fig 4, E). In summary, C03-H1L2 and C03-H2L2 significantly outperformed omalizumab in terms of IgE removal in both short- and long-term exposure experiments, demonstrating their potency to disrupt IgE from its high-affinity receptor in a time- and concentration-dependent manner.

**FIG 5.**
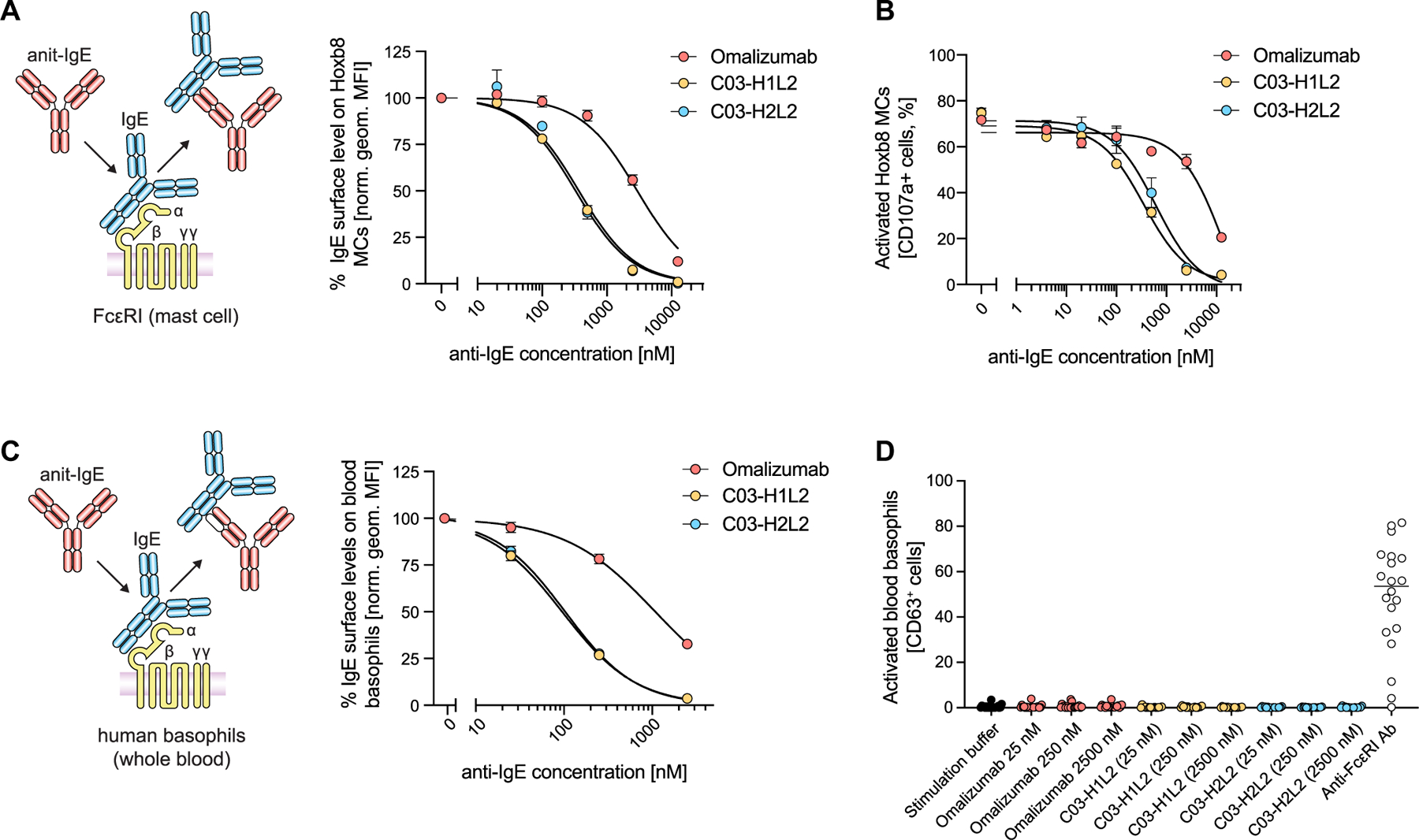
Cellular disruption kinetics and spontaneous activation assessment of anti-IgE antibodies. **(A)** Disruption of preformed IgE:FcεRIα complexes from the surface of Hoxb8 MCs by different concentrations (0.0008–12.5 μM) of omalizumab *(red)*, C03-H1L2 *(orange)*, and C03-H2L2 *(blue)* full-length IgGs over 20 hours of incubation as assessed by flow cytometry. **(B)** Quantification of percentage activated Hoxb8 MCs on subsequent antigen stimulation. **(C)** Whole blood of voluntary donors was incubated for 20 hours with 3 concentrations (25 nM, 250 nM, and 2500 nM) of omalizumab (n = 20), C03-H1L2 (n = 16), and C03-H2L2 (n = 16) full-length IgGs. Remaining cell surface IgE was quantified by flow cytometry. **(D)** Spontaneous activation of basophil on 30 minutes of exposure to anti-IgE antibodies and anti-FcεRIα (positive control) was measured by flow cytometry. Data of technical duplicates are shown as mean± SEM **(A** and **B)**. Data of 16 to 20 biological replicates is shown as mean ± SEM **(C)**. Data were normalized to untreated control levels, and binding curves were fitted using a nonlinear regression model **(A** and **C)**. *norm. geom. MFI*, Normalized geometric mean fluorescent intensity.

To further assess IgE dissociation efficacy in a cellular context, we presensitized Hoxb8 MCs with a fixed amount of recombinant human NIP-specific IgE (3 nM JW8-IgE), added increasing concentrations of anti-IgE antibodies (0.0008–12.5 μM) for 20 hours, and quantified the remaining cell surface IgE by flow cytometry (Fig 5, A; Fig E3, A, in the Online Repository at www.jacionline.org). Compared with omalizumab, both C03 variants showed an approximately 9-fold improved disruptive potency (DC_50_ C03-H1L2: 329 nM < C03-H2L2: 369 nM < omalizumab: 2951 nM). When we subsequently challenged the anti-IgE-treated Hoxb8 MCs with a fixed concentration of NIP_22_-BSA, the antigen-mediated activation was inhibited in an anti-IgE dose-dependent manner. The results corresponded well to the active removal of IgE from the cell surface and showed that C03-H1L2 and C03-H2L2 were superior inhibitors of antigen-mediated activation compared with omalizumab (Fig 5, B; Fig E3, A). As previously reported, the insertion of flexibility between the variable and constant region of the light and heavy chains of both C03 variants also enhanced disruptive potency compared with the parental C03 antibody (Fig E3, B).

To confirm these findings in a more physiologically relevant system, we additionally incubated whole blood of 20 human subjects with various concentrations of anti-IgE antibodies for 20 hours *in vitro* and measured the remaining cell surface IgE on primary human basophils by flow cytometry (Fig 5, C; Fig E3, C). Again, both C03 antibodies showed an approximately 10.5-fold decrease in the half-maximal disruptive concentration compared with omalizumab (DC_50_ C03-H1L2: 95 nM < C03-H2L2: 103 nM < omalizumab: 1085 nM). C03-H1L2 and C03-H2L2 both reached baseline levels (ie, no more surface IgE present) in both cellular experiments, whereas omalizumab did not result in complete IgE desensitization at the used concentrations. Importantly, none of the antibodies induced spontaneous basophil activation as assessed by percentage of CD63-positive basophils, whereas the majority of donor samples showed activation by an internal receptor cross-linking control antibody (Fig 5, D; Fig E3, B).

### High-affinity omalizumab variants rapidly desensitize allergic effector cells in a mouse model of systemic anaphylaxis

**FIG 6.**
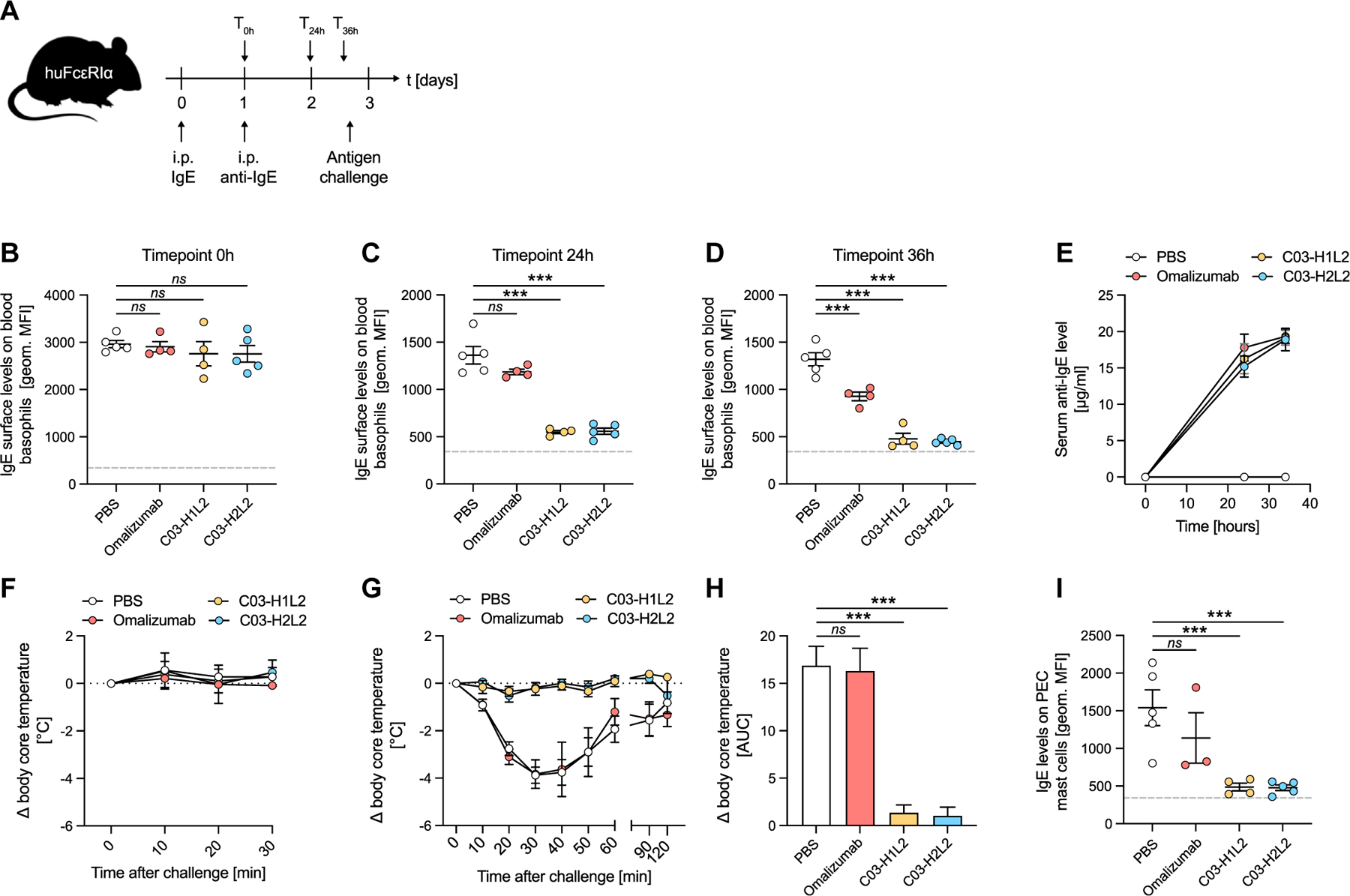
Anti-IgE-mediated inhibition of passive systemic anaphylaxis *in vivo*. **(A)** Experimental layout. Mice transgenic for human FcεRIα (n = 4–5 per group) were sensitized with JW8-IgE and 24 hours later treated with different anti-IgE antibodies. NIP_22_-BSA challenge was performed 36 hours after anti-IgE treatment. IgE surface levels on blood basophils were assessed at time point 0 hours **(B)**, time point 24 hours **(C)**, and time point 36 hours **(D)** by flow cytometry and displayed as geometric mean fluorescent intensity. **(E)** Plasma anti-IgE levels were measured by ELISA at indicated time points. **(F)** Core body temperature after anti-IgE treatment was assessed for 30 minutes and displayed as Δ°C body core temperature. **(G)** Core body temperature of mice is shown as Δ°C, **(H)** along with the area under the curve. **(I)** IgE surface level peritoneal MCs were assessed after antigen challenge by flow cytometry and displayed as geometric mean fluorescent intensity of indicated groups. Statistical significance was calculated by 1-way ANOVA with Tukey multiple comparisons against PBS treatment. ***P < .001. *AUC*, Area under the curve; *huFcεRI*α, human FcεRIα; *i.p.*, intraperitoneal; *norm. geom. MFI*, normalized geometric mean fluorescent intensity; *ns*, not significant; *T0, T24, and T36*, time points 0, 24, and 36 hours.

As we observed efficient dissociation of IgE:FcεRIα complexes with C03 variants *in vitro* and *ex vivo* on allergic effector cells, we next sought to investigate how the anti-IgE antibodies compare in a passive systemic anaphylaxis model using human FcεRIα transgenic mice (Fig 6, A).^36^ The mice received an intraperitoneal injection of recombinant human NIP-specific JW8-IgE (20 μg) to systemically sensitize allergic effector cells. The next day, they were treated with omalizumab, C03-H1L2, C03-H2L2 (200 μL at 10 μM), or PBS control for 36 hours. Subsequently, the mice were challenged with NIP_22_-BSA antigen (200 μg) to induce systemic anaphylaxis. Blood samples were collected before anti-IgE antibody administration (time point 0 hours), 1 day after anti-IgE administration (time point 24 hours), and before antigen challenge (time point 36 hours). At each time point, we quantified basophil cell surface IgE levels by flow cytometry and assessed the serum drug levels by ELISA. Basophil IgE levels were evenly distributed at time point 0 hours in all groups as measured by flow cytometry (Fig 6, B; Fig E4, A, in the Online Repository at www.jacionline.org). C03 antibodies removed the majority of basophil cell surface IgE within 24 hours (Fig 6, C) and reached nearly complete desensitization at time point 36 hours (Fig 6, D); however, for omalizumab it took 36 hours to observe a difference in IgE cell surface levels compared with mice treated with PBS control. Serum drug levels were similar for all anti-IgE antibodies and reached 15 to 20 μg/mL at time point 36 hours (Fig 6, E). As a readout for systemic anaphylaxis, we measured changes in body core temperature directly after anti-IgE antibody administration for 30 minutes and on antigen challenge over 2 hours. No change in body core temperature was observed for any of the C03 antibodies after systemic administration (Fig 6, F). On antigen challenge, however, both PBS control– and omalizumab-treated mice underwent pronounced systemic anaphylaxis manifesting as a loss of body core temperature (Fig 6, G and H). In contrast, C03-H1L2- and C03-H2L2-treated groups were fully protected against antigen challenge and showed no change in body core temperature, suggesting complete desensitization within 36 hours of treatment. Quantification of surface IgE levels on peritoneal MCs after antigen challenge confirmed the basophil results. C03-H1L2 and C03-H2L2 treatment removed almost all IgE from the cells, whereas there was still a considerable amount of IgE present on peritoneal MCs from mice treated with omalizumab or PBS control as measured by flow cytometry (Fig 6, I; Fig E4, B). Together, these results corroborate our previous finding that omalizumab is a “weak dissociator” of IgE:FcεRIα complexes with slow disruptive kinetics,^19^ whereas the presented C03 antibody variants remained safe and rapidly induced systemic desensitization of allergic effector cells *in vivo*.

## DISCUSSION

In this study, we investigated the structure-activity relationship for the 2 newly described flexible high-affinity omalizumab variants C03-H1L2 and C03-H2L2 and compared them with the therapeutic anti-IgE mAb omalizumab. We primarily focused on the assessment of their binding characteristics (ie, neutralization of IgE binding to FCεRI and CD23, IgE:IgG immune complex formation, and IgE:FcεRI complex disruption) and how these relate to the inhibition of allergic effector cell activation.

As C03-H1L2 and C03-H2L2 were engineered based on omalizumab, the binding epitope of all 3 antibodies on IgE remained highly conserved. However, their binding kinetics for free IgE substantially differed in that the C03 antibodies showed faster on-rates and slower off-rates. Overall, the affinities of monovalent C03-H1L2 and C03-H2L2 Fab fragments were approximately 10-fold higher for human IgE and approximately 4.5-fold higher for macaque IgE than Fab fragments of omalizumab. Importantly, the observed differences in affinity had no major impact on binding stoichiometry or anti-IgE:IgE complex formation. Depending on the molar ratio, all 4 anti-IgE antibodies formed 1:1 to maximally 4:4 complexes with the highest abundance of 3:3 complexes at equimolarity. This was consistent with previous reports for omalizumab.^18,37^ Importantly, we did not identify any formation of high-molecular-weight immune complexes that could potentially pose a problem *in vivo*.

The presented data suggest that nontraditional engineering of Fab elbows, through insertion of 1 or 2 Gly residues between the variable and constant region of the heavy and light chain, improved the dissociative capacity of the C03 antibodies, while only modestly impacting the antibodies stability and affinity to free IgE. Our data further indicate, that although there is a clear relationship between active dissociation and monomeric binding affinity, improvements in free IgE neutralization owing to improved affinity become evident only at very low IgE concentrations. As with all competitive inhibitors, neutralization efficiency is primarily driven by affinity once the concentration of the target is similar to the K_D_ of the inhibitor. However, additional inhibitor is required to neutralize any stoichiometric excess of the target. Anti-IgEs display picomolar avid binding and form multivalent complexes, and the goal of omalizumab therapy is to administer a minimum dose sufficient to reduce free IgE levels to approximately 25 ng/mL (10 kU/L), a threshold associated with clinical efficacy.^38^ To achieve this, omalizumab must be administered at a substantial molar excess, which is typically 15 to 20 times greater than the baseline total IgE concentration. As IgE levels rise, the required omalizumab dose increases disproportionately, making suppression of very high IgE levels impractical within approved dosing limits. Our data suggest that pure affinity improvements of anti-IgE would enhance neutralization only modestly at normal total IgE levels (1.5–114 kU/L^39^), but could help further neutralize free IgE binding at low IgE levels (eg, the therapeutic target free IgE concentrations of <10 kU/L or approximately 120 pM). As such, we would expect that the high-affinity C03 variants could potentially be used to treat patients with higher IgE levels than 700 kU/L.^40^ On a separate note, the presence of omalizumab compromises accurate quantitation of total and free IgE levels in serum samples of treated patients by certain diagnostic assays.^41^ However, these values represent important biomarkers to interpret treatment efficacy or to optimize dosing regimen in the clinical setting. Preanalytic depletion of IgE:drug complexes or generation of anti-IgE antibodies with accelerated systemic clearance^42^ could be helpful in this context. Incorporation of a targeted Fc mutation (ie, S267E/L328F)^43^ to the described C03 variants increasing their affinity to FcγRIIb could be an interesting approach to achieve faster complex clearance. These observations have important implications for the design of novel anti-IgE antibodies and suggest that improved efficacy of anti-IgE antibodies cannot be driven by modulating affinity alone.

We previously highlighted that the ability to actively remove IgE from its high-affinity receptor FCεRI on allergic effector cells was a largely ignored mode of action in the development and use of omalizumab.^44^ Whereas omalizumab can certainly be classified as a “weak dissociator”^19^ of IgE:FcεRI complexes based on its low efficacy to remove IgE from FcεRI, there is accumulating evidence that this molecular mechanism might still be of important clinical relevance—especially as the high-affinity anti-IgE mAb ligelizumab,^45^ which completely lacks this disruptive activity,^15^ failed to demonstrate superiority over omalizumab in clinical trials to date.^46^ Here, we further demonstrated that the flexible high-affinity omalizumab variants C03-H1L2 and C03-H2L2 not only feature tighter binding to free IgE, but also show substantially increased disruptive potency in both molecular and cellular assays. Notably, both C03 antibodies completely desensitized basophils and MCs *ex vivo* in less than 1 day in the micromolar concentration range. Although the used molecular assays including ELISA and SPR measurements are useful for a standardized characterization of binding or disruption mechanisms, it is important to note that cellular assays with basophils or MCs are more reflective of the *in vivo* situation.

We also showed that the described disruptive activity of anti-IgE antibodies has remarkable *in vivo* relevance at serum concentrations similar to those observed in omalizumab clinical studies. In the passive systemic anaphylaxis mouse model, blood basophils and peritoneal MCs were completely desensitized within less than 36 hours in the C03 antibody variant treatment groups. Importantly, the measured decrease of IgE levels on the cell surface occurred faster than the expected cellular turnover *in vivo* (ie, 5 days for basophils^47^; 3–4 weeks for MCs^48^). These results confirmed that the observed effect was due to treatment-induced IgE removal and not to IgE neutralization activity. The fact that only C03-H1L2- and C03-H2L2-treated mice were protected from anaphylaxis on antigen challenge further indicated that omalizumab treatment did not result in sufficient IgE removal within the experimental time frame. These specific conditions were deliberately chosen, as there are certain clinical situations in which a fast desensitization would be of great clinical benefit, such as in severe drug allergies or allergen immunotherapies.

In summary, this study demonstrates that various molecular binding aspects of omalizumab could be improved to potentially further enhance treatment efficacy, while conserving important features including epitope specificity, stoichiometry, and capacity to inhibit CD23 binding. Our data confirmed that both increasing Fab flexibility and enhancing antibody affinity help to improve disruptive capacity, which considerably accelerates desensitization of allergic effector cells *in vivo*. The development of next-generation anti-IgE antibodies with optimized binding characteristics is a challenging task, and it will be interesting to see whether the presented findings will facilitate this process in the near future.

## Supplementary Material

1

2

3

4

5

## Figures and Tables

**TABLE I.** Binding kinetics of omalizumab variants to recombinant human and macaque IgE

Anti-IgE antibody	Association k_a_ (M^−1^s^−1^)	Dissociation k_d_ (s^−1^)	Affinity K_D_(M)
	**Human IgE**		
Omalizumab IgG	9.32 × 10^5^	5.54 × 10^−5^	5.94 × 10^−11^
Omalizumab Fab	4.20 × 10^5^	6.14 × 10^−3^	1.46 × 10^−8^
C03 IgG	1.03 × 10^6^	3.66 × 10^−5^	3.55 × 10^−11^
C03 Fab	1.28 × 10^6^	1.38 × 10^−3^	1.08 × 10^−9^
C03-H1L2 IgG	9.31 × 10^5^	2.69 × 10^−5^	2.88 × 10^−11^
C03-H1L2 Fab	7.34 × 10^5^	1.05 × 10^−3^	1.43 × 10^−9^
C03-H2L2 IgG	7.70 × 10^5^	2.91 × 10^−5^	3.78 × 10^−11^
C03-H2L2 Fab	7.10 × 10^5^	1.08 × 10^−3^	1.52 × 10^−9^
	**Macaque IgE**		
Omalizumab IgG	1.57 × 10^6^	8.52 × 10^−5^	5.43 × 10^−11^
Omalizumab Fab	5.04 × 10^5^	8.11 × 10^−3^	1.61 × 10^−8^
C03 IgG	1.19 × 10^6^	9.90 × 10^−6^	8.33 × 10^−11^
C03 Fab	1.96 × 10^6^	6.41 × 10^−3^	3.27 × 10^−9^
C03-H1L2 IgG	1.17 × 10^6^	9.46 × 10^−5^	8.08 × 10^−11^
C03-H1L2 Fab	1.24 × 10^6^	4.11 × 10^−3^	3.32 × 10^−9^
C03-H2L2 IgG	1.10 × 10^6^	9.57 × 10^−5^	8.72 × 10^−11^
C03-H2L2 Fab	1.18 × 10^6^	4.15 × 10^−3^	3.53 × 10^−9^

## Clinical implications:

C03-H1L2 and C03-H2L2 represent promising candidates for advancing allergy treatment, as they lay the conceptual basis to improve the therapeutic efficacy and speed of anti-IgE approaches.

## Abbreviations used

DC_50_: Half-maximal disruptive concentration
Fab: Fragment antigen-binding
Gly: Glycine
IC_50_: Half-maximal inhibitory concentration
k_a_: Association constant
k_d_: Dissociation constant
K_D_: Equilibrium dissociation constant
MC: Mast cell
NIP: 3-Nitro-4-hydroxy-5-iodophenylacetyl
SEC: Size exclusion chromatography
SPR: Surface plasmon resonance
VH: Variable heavy chain
VL: Variable light chain
