# Supplementary figures and images for: High-affinity omalizumab variants with optimized disruptive potency prevent anaphylaxis in vivo
Source: J Allergy Clin Immunol. Author manuscript; Available in PMC 2026 Jun 14. (PMC13264800; doi:10.1016/j.jaci.2025.05.028)

Figure E1

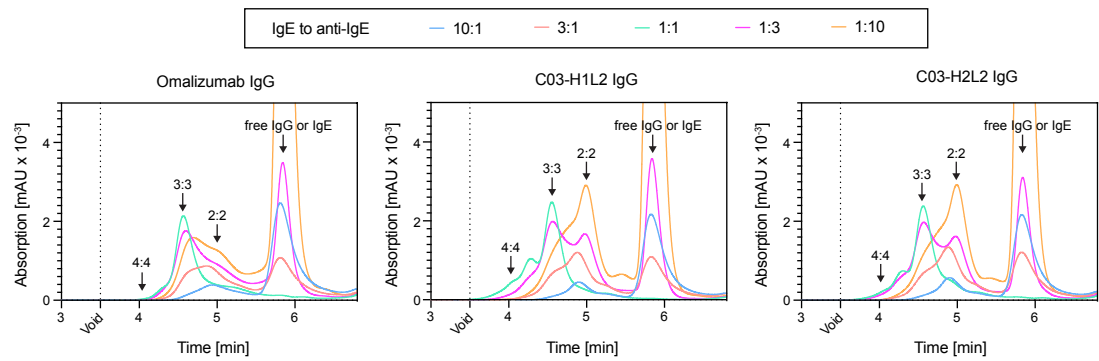

Supplement: 1 [file NIHMS2177603-supplement-1.pdf]

Figure E2

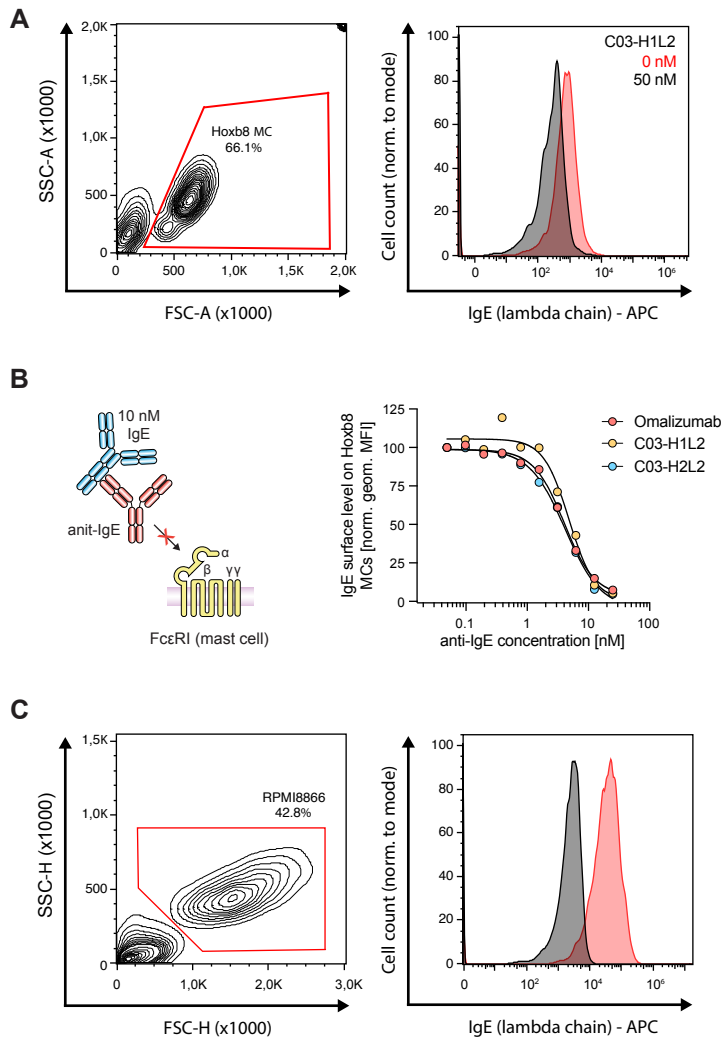

Supplement: 2 [file NIHMS2177603-supplement-2.pdf]

Figure E3

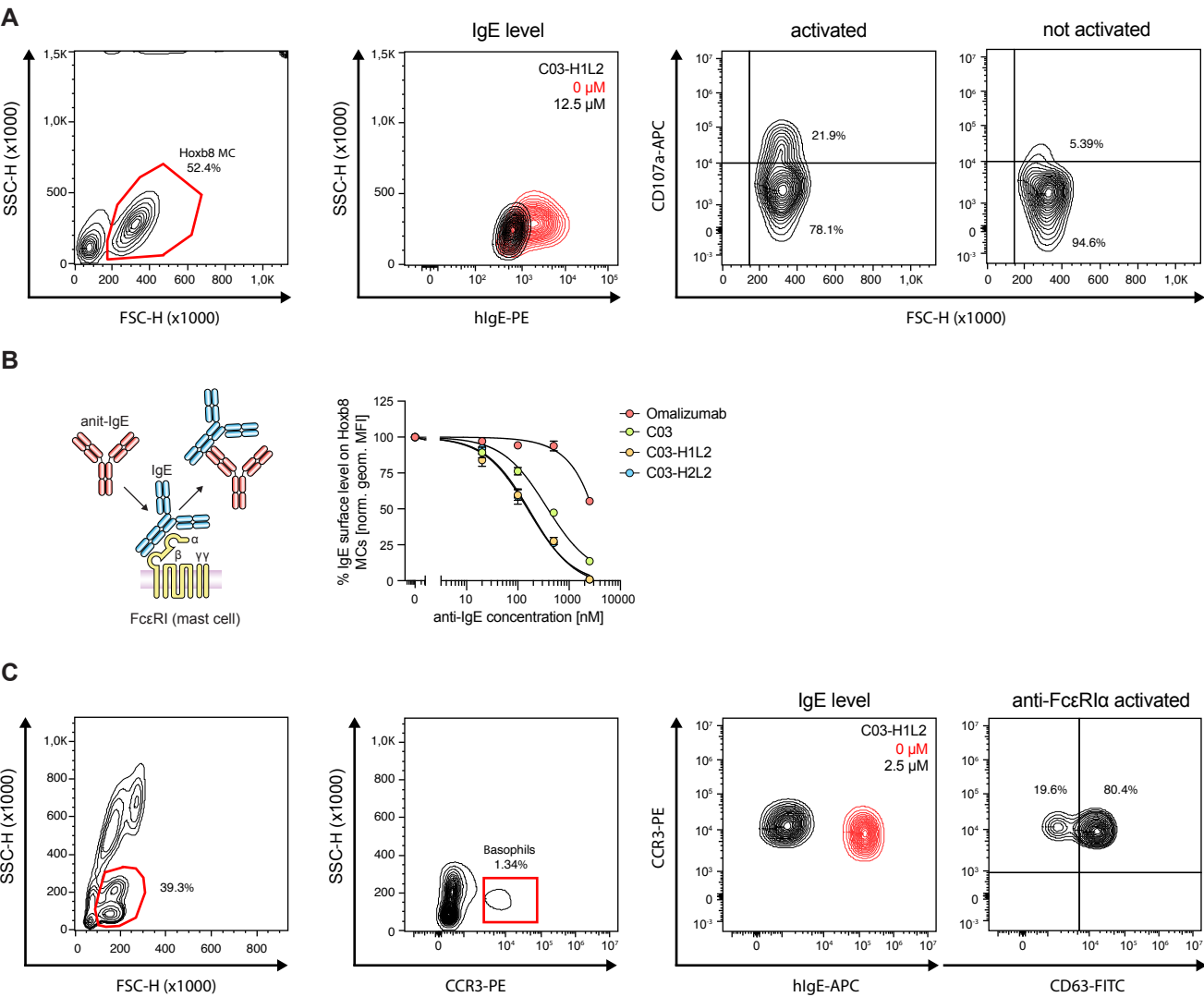

Supplement: 3 [file NIHMS2177603-supplement-3.pdf]

Figure E4

**A**

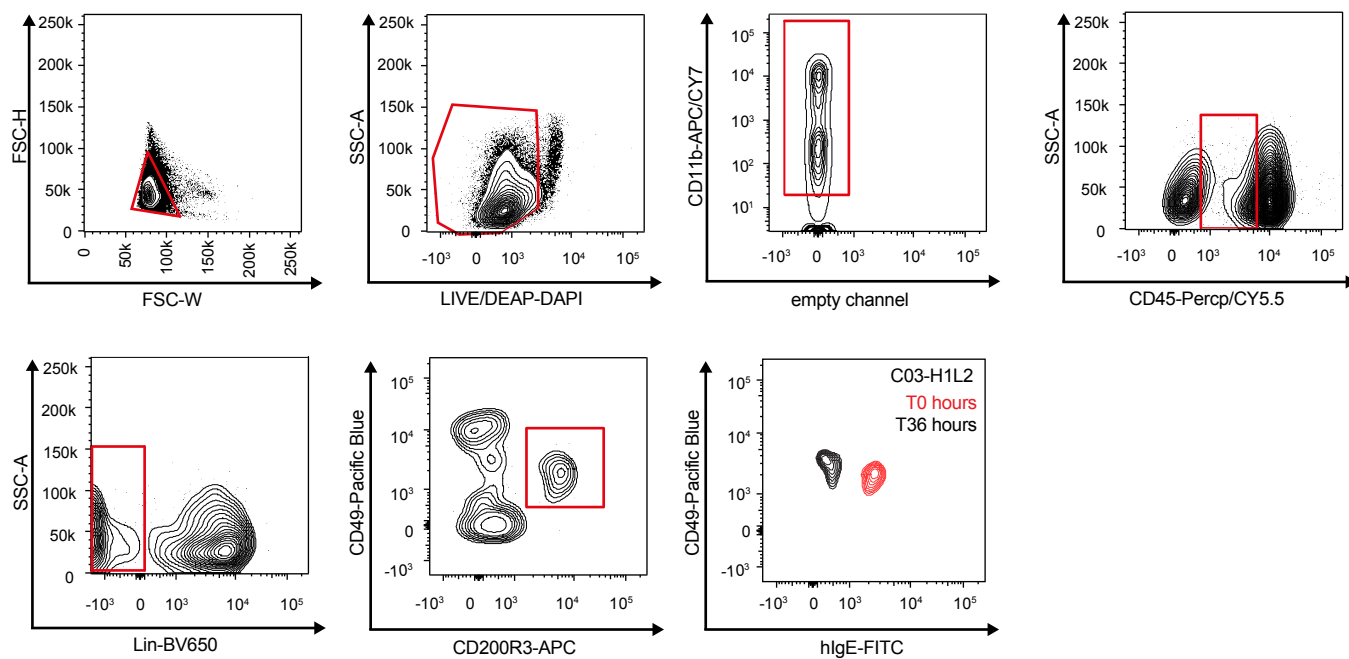

**B**

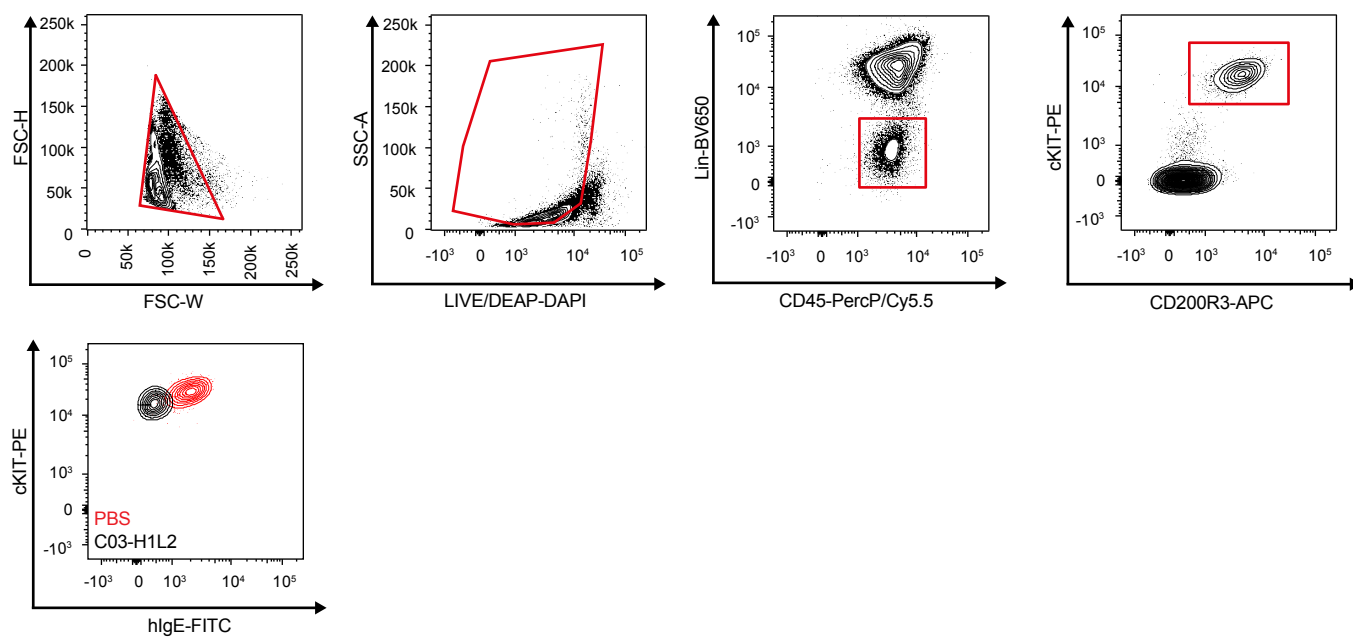

Supplement: 4 [file NIHMS2177603-supplement-4.pdf]
