# Supplementary material for: High-affinity omalizumab variants with optimized disruptive potency prevent anaphylaxis in vivo
Source: J Allergy Clin Immunol. Author manuscript; Available in PMC 2026 Jun 14. (PMC13264800; doi:10.1016/j.jaci.2025.05.028)
Supplement: 5 [file NIHMS2177603-supplement-5.docx]

Online Repository

Title: High-affinity omalizumab variants with optimized disruptive potency prevent anaphylaxis *in vivo*

**Authors:** Daniel Brigger, PhD^1,2,†^, Pascal Guntern, PhD^1,2,†^, Luke F. Pennington, PhD^3^_,_ Robin van Brummelen, MSc^1,2^, Theodore S. Jardetzky, PhD^4^ and Alexander Eggel, PhD^1,2*^

**Affiliations:**

^1^Department of BioMedical Research, University of Bern, Bern, Switzerland

^2^Department of Rheumatology and Immunology, University Hospital Bern, Bern, Switzerland

^3^Excellergy Inc, Palo Alto, California 94306, USA.

^4^Department of Structural Biology, Stanford University School of Medicine, Stanford, California 94305, USA.

*Corresponding author: Prof. Alexander Eggel, University of Bern, DBMR, Murtenstrasse 28, LPM, 3008 Bern, Switzerland; Phone: +41 31 684 04 30; Email: alexander.eggel@unibe.ch

† These authors contributed equally to this work.

**METHODS**

***Recombinant proteins and antibodies:*** Anti-IgE antibodies as well as human and macaque trastuzumab-IgE were recombinantly produced based on available sequence information (Curia, NY, USA). Recombinant Sus11-IgE (hybridoma), recombinant monoclonal humanized 3-nitro-4-hydroxy-5-iodophenylacetyl (NIP)-specific JW8-IgE, expressing a mouse lambda chain and FcεRIα-HSA fusion protein were purchased from NBS-C BioScience (Vienna, Austria). Recombinant human CD23 and human epidermal growth factor receptor 2 (Her2) were purchased (R&D Systems, Minneapolis, MN, USA). Recombinant human IL-3 was purchased from Peprotech (London, UK). NIP_22_-BSA as well as NIP_24_-BSA was purchased from LGC Biosearch Technologies (Teddington, UK).

***Protein interaction measurements with SPR.***

To determine binding kinetics of anti-IgE antibodies/fragments for recombinant human and macaque IgE, 2500 RU recombinant Her2 cancer antigen (R&D Systems) were immobilized on Fc2 in acetate buffer at pH 4.0. A concentration of 35 nM human or macaque trastuzumab-IgE (anti-Her2-IgE) was subsequently captured for 120 s to reach a response of ~100 RU. Various concentrations (1.56–50 nM) of anti-IgE antibodies/fragments were injected for 120 s and the dissociation was measured for 600 s under constant buffer flow. After each run, the chip surface was regenerated with 40 mM NaOH and reloaded with trastuzumab-IgE.

To determine disruptive efficacy of anti-IgE antibodies, 1000 RU recombinant FcεRIα-HSA fusion protein (NBS-C BioScience) were immobilized on Fc2 in acetate buffer at pH 4.0. 500 RU of recombinant HSA (Sigma-Aldrich, MA, USA) in acetate buffer pH 4.0 was immobilized on Fc1 as a control. A concentration of 35 nM Sus11-IgE (NBS-C BioScience) was subsequently captured for 60 s to reach a response of >200 RU. Various concentrations (250–2000 nM) of anti-IgE antibodies were injected for 540 s and the dissociation was measured for 180 s under constant buffer flow. After each run, the chip surface was regenerated with 40 mM NaOH and reloaded with Sus11-IgE. To calculate the percentage of removed IgE, RU at start of injection was set into relation to RU at the end of injection (100 / RU_start_ x RU_end_).

To assess disruptive efficacy of anti-IgE antibodies over longer injection time, 1000 RU of recombinant FcεRIα-HSA fusion protein (NBS-C BioScience) were immobilized on flow cell 2 at pH 4.0. A blank immobilization was performed on flow cell 1. A concentration of 30 nM Sus11-IgE was subsequently captured for 120 s to reach a response > 200 RU. Anti-IgE antibodies were injected at 1 µM for 42 times 540 s with a dissociation time of 180 s between each injection under constant buffer flow. At the end of each run the chip surface as regenerated with 50 mM NaOH and reloaded with Sus11-IgE. As shown in the respective sensorgrams, the receptor retained full IgE-binding capacity over the course of the entire experiment (including ≥ 20 rounds of regeneration).

***Immune complex analysis.*** IgE and anti-IgE molecules were combined in a 100 μL volume of PBS at room temperature for one hour. The final concentration of recombinant human trastuzumab-IgE was 0.1 mg/mL in all experiments and the concentration of the anti-IgE was varied according to the following molar IgE:anti-IgE ratios: 10:1, 3:1, 1:1, 1:3, and 1:10. Immune complexes were directly analyzed by SEC-MALS-RI except samples with molar ratios of 10:1 and 3:1, which were concentrated 2-fold using 3k MWCO centrifugal filters prior to injection. Immune complexes were analyzed by analytical size exclusion chromatography (SEC) coupled to a μDAWN multi-angle light scattering (MALS) detector and an Optilab UT-rEX refractive index (RI) detector (Wyatt Technology). Chromatography was performed using an Agilent 1260 Infinity II Bio-inert system on an Acquity UPLC BEH450 SEC column (2.5 μm, 4.6 x 150 mm). The column was equilibrated with PBS at a flow rate of 0.3 mL/min at 25 °C. Data collection and SEC-MALS- RI analysis were performed with Astra 7.3 software (Wyatt Technology). The refractive index of the solvent was 1.331 and the refractive index increment (dn/dc) used for all proteins was 0.185 mL/g.

***ELISAs.***

To assess anti-IgE-meditated blocking of IgE binding to CD23, recombinant human CD23 was immobilized at 60 nM on the plastic surface of a 96-half-well plate (Corning, NY, USA) by overnight incubation in PBS at 4 °C. The following the day the plate was blocked with PBS/0.15 % casein for two hours at room temperature (RT). IgE-antigen complexes were generated by incubating biotinylated recombinant monoclonal humanized JW8-IgE (NBS-C BioScience) at 240 nM with 240 nM NIP_24_-BSA (LGC Biosearch Technologies) in PBS/0.15 % casein for 30 minutes at RT. The IgE-antigen complexes were then mixed with a 1:3 serial dilution of the anti-IgE antibodies (240 – 0.3292 nM) and incubated for 30 minutes at RT prior to the incubation on the ELISA plate for one hour at RT while shaking. IgE binding to immobilized CD23 was detected by incubating the plate with Pierce™ Streptavidin Poly-HRP (Thermo Fisher Scientific, MA, USA) 1:3000 diluted in PBS/0.15 % casein followed by development with TMB (3,3′,5,5′-tetramethylbenzidine, Merck, Darmstadt, Germany). The reaction was stopped using 1 M sulfuric acid. Washing steps were performed using PBS and PBS/0.05 % Tween. Absorbance was measured at 450 nm.

To assess inhibition of IgE binding to FcεRI, human recombinant FcεRIα was immobilized at a concentration of 30 nM on the ELISA plate by overnight incubation at 4°C in PBS. Biotinylated JW8-IgE was incubated at a concentration of 0.04 nM with a 1:5 serial dilution of the anti-IgE antibodies (30 – 0.002 nM) for 30 minutes at RT prior to the incubation on the ELISA plate for one hour at RT while shaking. All following steps were performed as mentioned above.

The anti-IgE mediated disruption of IgE-FcεRI complexes was measured by ELISA using the same human recombinant FcεRIα immobilized at a concentration of 30 nM on the plastic surface of a 96-half-well plate by overnight incubation in PBS. After blocking, biotinylated Sus11-IgE was added to the plate at 0.1 nM in PBS/0.15% casein for one hour at RT while shaking. Unbound IgE was removed by washing the plate with PBS and PBS/0.05 % Tween. The IgE-FcεRIα complexes were then incubated with a 1:3 serial dilution of the anti-IgE antibodies (5000 – 0.76208 nM) in PBS/0.15% casein for twelve hours at RT while shaking. The remaining Sus11-IgE biotin was detected as described above.

To assess time-dependent disruption of IgE-FcεRIα complexes, immobilized recombinant human FcεRIα (30 nM) was incubated with 0.1 nM biotinylated Sus11-IgE for one hour at RT. Afterwards 5000 nM of the anti-IgE antibodies were incubated for 2, 4, 8, 12 or 24 hours. The remaining IgE was detected as described above. All plates were developed at the same time. Start of FcεRIα immobilization, Sus11-IgE and anti-IgE antibody incubation were planned accordingly. Absorbance values were normalized to untreated samples from timepoint zero.

Plasma anti-IgE antibody concentrations were determined using the IgG high sensitivity ELISA (Mabtech AB, Sweden) according to the manufacturer’s instructions with the single modification that we used 96-half-well plate (Corning, NY, USA). Optical density was measured using an ELISA reader at 450nm within 15 minutes.

***Cellular assays.*** RPMI8866, expressing high levels of human CD23, cells were cultured in RPMI+/+ medium composed of RPMI 1640 medium (Biochrome, Cambridge, UK) complemented with 10% Hyclone FCS (Fisher Scientiﬁc, NH, USA), penicillin 100 U/ml, 100 µg/ml streptomycin (100× penicillin/streptomycin, Merck, Darmstadt, Germany) and 10 mM HEPES buffer (stock-solution 1 M, Life Technologies, CA, USA). To assess anti-IgE antibody mediated blocking of IgE binding to CD23 on RPMI8866 cells, seven concentrations of the anti-IgE antibodies (0.003-50 nM) were mixed with 12.5 nM JW8-IgE and allowed to complex for 30 minutes. The mixture was added to RPMI 8866 cells and incubated for one hour at 37 °C/5% CO_2_, followed by staining with a fluorescently labeled anti-mouse-lambda light chain antibody to detect remaining cell bound JW8-IgE. Cells were then washed and resuspended in FACS buffer for acquisition.

Hoxb8 MCs were cultured in RPMI+/+ including ~10 ng/ml IL-3 and 4-OHT (i.e. tamoxifen). To differentiate progenitors into mature allergic effector cells, cells were washed twice in PBS and reseeded at 7.5 x 10^4^ cells/mL in the same medium lacking tamoxifen for 5 days. Cells were centrifuged at 250 x g for 5 minutes and resuspended in BMMC medium (RPMI+/+ including 1 mM Sodium Pyruvate, 4 mM L-Glutamine, 1x Non-Essential Amino acids, 50 µM Mercaptoethanol and 30 ng/ml recombinant murine IL-3). To sensitize the cells 50,000 cells were seeded in a 96-well plate and 3 nM JW8-IgE was added overnight to the cells in BMMC medium. The next day, cells were washed three times with PBS and 50 µl of anti-IgE antibodies in different concentrations (0.8-12500 nM) were added for 20 hours to measure IgE disruption. Cells were washed three times with FACS buffer and cells resuspended in staining mix containing a fluorescently labelled anti-human IgE antibody (clone IgE21) and kept on ice for 15 min. Cells were then washed once and resuspended in 200 µl FACS buffer for acquisition on a CytoFLEX device (Beckman Coulter, CA, USA). For the inhibition of IgE binding to FcεRIα-expressing Hoxb8 MCs by the anti-IgE antibodies cells were differentiated accordingly. Biotinylated Sus11-IgE was incubated at a concentration of 1 nM with different concentrations (0.03-25 nM) of the anti-IgE antibodies for 30 minutes at RT. These mixtures were then added to 50,000 seeded Hobx8 MCs and incubated for one hour at 37°C. Cells were washed three times with FACS buffer and cells resuspended in staining mix containing a fluorescently labeled anti-mouse-lambda light chain antibody to detect remaining cell-bound Sus11-IgE and kept on ice for 15 min. Cells were then washed once and resuspended in 200 µl FACS buffer for acquisition on a CytoFLEX device (Beckman Coulter, CA, USA).

Whole blood from voluntary donors was collected at the University Hospital Bern with approval from the local ethics committee of the Canton of Bern (KEK 2018-00204). Informed consent was obtained in accordance with the Declaration of Helsinki. Basophil activation test was performed using a modified protocol of BÜHLMANN Laboratory’s Flow CAST. Briefly, 50 µl whole blood were added to 100 µl stimulation buffer (containing recombinant IL-3) and 12 µl staining mix in a 96-well plate and incubated for 30 min at 37°C/5% CO_2_ with 50 µl of 4.24-fold concentrated anti-IgE antibodies (final concentration 2.5-2500 nM). Subsequently, cells were centrifuged at 600 x g for 5 minutes and supernatants discarded. The cell pellet was reconstituted in 200 µl of lysing reagent and erythrocytes were lysed for 7 min. The centrifugation and lysis step were repeated two times. Cells were resuspended in 50 µl FACS buffer containing a fluorescently labeled anti-IgE antibody and incubated for 15 min on ice. In a last stop, 150 µl FACS buffer was added and cells centrifuged at 600 x g for 5 minutes before discarding the supernatant and resuspending the cells in 200 µl FACS buffer. Samples were acquired on a CytoFLEX device (Beckman Coulter, CA, USA).

To calculate percentage of IgE removal, background MFI (autofluorescence of cells) was subtracted and MFI normalized to IgE levels of samples without anti-IgE treatment.

***Passive systemic anaphylaxis mouse model.*** Mice transgenic for human FcεRIα (huFcεRIα^tg^) on a mixed C57BL/6J–C57BL/6N background were kindly provided by Prof. J.-P. Kinet. All animal experimentation was approved by the local ethics committee of the Canton of Bern (authorization BE53/2021). huFcεRIα^tg^ were bred and housed in specific pathogen-free conditions at the University of Bern. Mice were passively sensitized intraperitoneally with 20 µg NIP-specific human JW8-IgE. The next day, 200 µl of anti-IgE antibody variants were injected intraperitoneally at a concentration of 10 µM. Mice were challenged 36 hours post anti-IgE treatment with 200 µg NIP_22_-BSA (LGC Biosearch Technologies). Body core temperature was assessed with MiniTemp (Vectronic Services Ltd) for 2 hours. The concentrations of IgE, anti-IgE and antigen were chosen based on previous experience with this mouse line as well as in vitro assay informed estimations. Blood was drawn at indicated time points to assess IgE surface levels on blood basophils using flow cytometry and to measure anti-IgE antibody concentrations in the plasma fraction. Temperature loss (Δ core body temperature) is represented for each time point after subtracting baseline temperature and area under the curve has been calculated using prism. Mice were sacrificed and peritoneal cavity (i.e. PEC) collected for further analysis.

***FACS analysis.*** Single cell suspensions were prepared using ACK lysis buffer, 70 µm cell strainer (Falcon) and blocked using CD16/32 (Biolegend) blocking antibodies and measured by FACS (BD FACS sorp LSR II) and analysed using FlowJo software. Peritoneal mast cells and blood basophils were identified as live using DAPI (Thermo Fisher Scientific) and the following antibody combination: anti-mouse CD3 (BD Bioscience; 145-2c11), anti-mouse NK1.1 (BD Bioscience; PK136) all biotinylated and were detected using streptavidin-BV650 (Biolegend). Anti-mouse CD45-PercpCy5.5 (Biolegend; 30-F11), anti-mouse c-KIT (eBioscience; 2B8), anti-mouse CD200R3 (Biolegend; Ba13), anti-mouse CD49b (Biolegend; DX5) and anti-human IgE (eBioscience;IgE21). Blood basophils were identified as: live, Lin^-^ (CD19, NK1.1, CD4/CD8), CD45^+^, CD200R3^+^, CD49b^+^ and IgE^+^. PECS were identified as: Lin^-^ (CD19, NK1.1, CD4/CD8), CD45^+^, c-Kit^+^, CD200R3^+^ and IgE^+^.

**FIGURE LEGENDS**

**Figure E1. Size determination of IgE:anti-IgE complexes.** IgE:anti-IgE immune complexes were determined with omalizumab, C03-H1L2 and C03-H2L2 full-length IgG at molar ratios of 10:1, 3:1, 1:1, 1:3, and 1:10 and were analyzed by size exclusion chromatography. Histograms show the protein absorption peaks at different time points. Different colors of the histograms refer to the indicated molar ratios. Expected binding stoichiometries (1:1 to 4:4) based on calculated molecular weights (kDa) are annotated in the histograms.

**Figure E2. Flow cytometry gating strategies for quantification of IgE-binding inhibition to FcεRIα or CD23. a** Hoxb8 mast cells were identified based on FSC and SSC gating and the amount of cell surface JW8-IgE was quantified by anti-lambda chain staining. The IgE levels for the 0 nM (red) versus 50 nM (black) C03-H1L2 treatment conditions are shown as representative histograms. **b** Normalized inhibition of 10nM IgE binding to FcεRIα on Hoxb8 mast cells by omalizumab (red), C03-H1L2 (orange) and C03-H2L2 (blue) full-length IgGs measured by flow cytometry. **c** RPMI8866 B-cell myeloma cells were identified based on FSC and SSC gating and the amount of cell surface JW8-IgE was quantified by anti-lambda chain staining. The IgE levels for the 0 nM (red) versus 50 nM (black) C03-H1L2 treatment conditions are shown as representative histograms.

**Figure E3. Flow cytometry gating strategies for quantification of IgE disruption from FcεRIα. a** Hoxb8 mast cells were identified based on FSC and SSC gating and the amount of cell surface JW8-IgE was quantified by anti-human IgE staining. The IgE levels for the 0 nM (red) versus 12.5 µM (black) C03-H1L2 treatment conditions are shown as representative contour plots. Activation was measured by anti-CD107a staining. **b** Disruption of pre-formed IgE:FcεRIα complexes from the surface of Hoxb8 mast cells by different concentrations (20-2500 nM) omalizumab (red), C03 (green), C03-H1L2 (orange) and C03-H2L2 (blue) full-length IgGs over 20 hours incubation as assessed by flow cytometry. **c** Human basophils were identified based on FSC and SSC gating and CCR3 positivity. The amount of intrinsic IgE was quantified by anti-human IgE staining. The IgE levels for the 0 nM (red) versus 2.5 µM (black) C03-H1L2 treatment conditions are shown as representative contour plots. Activation was measured by anti-CD63 staining.

**Figure E4. Flow cytometry gating strategies for quantification of IgE levels on basophils and peritoneal mast cells in mice.**  **a** Mouse basophils were identified as singlets, live, CD11b^pos^, CD45^inter^, lin^neg^, CD49^pos^, CD200R3^pos^ cells. The amount of intrinsic IgE was quantified by anti-mouse IgE staining. The IgE levels for timepoint 0 hours (red) versus 36 hours of C03-H1L2 (black) treatment is shown as representative contour plot. **b** Mouse peritoneal mast cells were identified as singlets, live, lin^neg^, CD45^pos^, cKIT^pos^, CD200R3^pos^ cells. The amount of intrinsic IgE was quantified by anti-mouse IgE staining. The IgE levels for the PBS control (red) versus C03-H1L2 (black) treatment condition is shown as representative contour plot.

**TABLES**

**Table E1. Thermal stability of recombinantly produced anti-IgE antibodies.**

| Anti-IgE antibody | Concentration (mg/ml) | T_m_ (°C) | T_agg_ (°C) |
| --- | --- | --- | --- |
| Omalizumab | 10 | 67 | 79 |
| C03 IgG | 10 | 67 | 75 |
| C03-H1L2 IgG | 10 | 63 | 65 |
| C03-H2L2 IgG | 10 | 62 | 64 |

T_m_: melting temperature; T_agg_: Thermal aggregation
